# Obesity

**Published:** 1914-09

**Authors:** 


**Obesity.** Andre Philibert, Progres Med., July 4, 1914. The author recognizes the following types: Obesity by excess of nutrition; by default of assimilation or lymphatic obesity; associated with diabetes; associated with gout or renal lithiasis; due to insufficience of glands of internal secretion, including thyroid obesity, that of genital type which may be congenital and associated with thyroid failure, that due to hypohysieal failure, that due to suprarenal failure and even a pluriglandular type. In the treatment of the type due to excess of nutrition, he emphasizes the importance of moderate diminiution of calories, moderate exercise, so as not to cause too much hunger, and even advocates extra meals to take the edge off the appetite for hearty meals. Hydrotherapy is discussed in the usual way. In regard to the puzzling type due to deficient assimilation, in habitual, light eaters, he mentions Labbe's observation that there is apt to be retention of chlorids and advises a low ration of chlorids. He emphasizes the fact that thyroid medication should be used only when there is deficient thyroid secretion although he does not offer suggestions to help those of us who fully appreciate this fact but are often in doubt as to how to determine the fundamental fact in obscure cases. In obesity associated with diabetes, gout, lithiasis, etc., the diet should be regulated according to the associated condition rather than with a view to reducing weight directly. As to obesity due to failure of internal secretions other than the thyroid, while he mentions ovarion opotherapy, he is obviously in the state of many of us, unable to offer thoroughly satisfactory methods of treatment.

